# Expression of an endotoxin-free S-layer/allergen fusion protein in gram-positive *Bacillus subtilis *1012 for the potential application as vaccines for immunotherapy of atopic allergy

**DOI:** 10.1186/1475-2859-10-6

**Published:** 2011-02-10

**Authors:** Nicola Ilk, Christian-Thomas Schumi, Barbara Bohle, Eva Maria Egelseer, Uwe B Sleytr

**Affiliations:** 1 Department of NanoBiotechnology, University of Natural Resources and Life Sciences (BOKU), Vienna, A-1190 Vienna, Austria; 2 Christian Doppler Laboratory for Immunomodulation, Department of Pathophysiology and Allergy Research, Center of Pathophysiology, Infectiology & Immunology, Medical University of Vienna, A-1090 Vienna, Austria

## Abstract

**Background:**

Genetic fusion of the major birch pollen allergen (Bet v1) to bacterial surface-(S)-layer proteins resulted in recombinant proteins exhibiting reduced allergenicity as well as immunomodulatory capacity. Thus, S-layer/allergen fusion proteins were considered as suitable carriers for new immunotherapeutical vaccines for treatment of Type I hypersensitivity. Up to now, endotoxin contamination of the fusion protein which occurred after isolation from the gram-negative expression host *E. coli *had to be removed by an expensive and time consuming procedure. In the present study, in order to achieve expression of pyrogen-free, recombinant S-layer/allergen fusion protein and to study the secretion of a protein capable to self-assemble, the S-layer/allergen fusion protein rSbpA/Bet v1 was produced in the gram-positive organism *Bacillus subtilis *1012.

**Results:**

The chimaeric gene encoding the S-layer protein SbpA of *Lysinibacillus sphaericus *CCM 2177 as well as Bet v1 was cloned and expressed in *B. subtilis *1012. For that purpose, the *E. coli-B. subtilis *shuttle vectors pHT01 for expression in the *B. subtilis *cytoplasm and pHT43 for secretion of the recombinant fusion protein into the culture medium were used. As shown by western blot analysis, immediately after induction of expression, *B. subtilis *1012 was able to secret rSbpA/Bet v1 mediated by the signal peptide amyQ of *Bacillus amyloliquefaciens*. Electron microscopical investigation of the culture medium revealed that the secreted fusion protein was able to form self-assembly products in suspension but did not recrystallize on the surface of the *B. subtilis *cells. The specific binding mechanism between the N-terminus of the S-layer protein and a secondary cell wall polymer (SCWP), located in the peptidoglycan-containing sacculi of *Ly. sphaericus *CCM 2177, could be used for isolation and purification of the secreted fusion protein from the culture medium. Immune reactivity of rSbpA/Bet v1 could be demonstrated in immunoblotting experiments with Bet v1 specific IgE containing serum samples from patients suffering birch pollen allergy.

**Conclusions:**

The impact of this study can be seen in the usage of a gram-positive organism for the production of pyrogen-free self-assembling recombinant S-layer/allergen fusion protein with great relevance for the development of vaccines for immunotherapy of atopic allergy.

## Background

S-layers are crystalline arrays of proteinaceous subunits representing the outermost cell envelope component in many bacteria and most archaea [1-3]. One of the most remarkable properties of isolated S-layer proteins is their capability to self-assemble in suspension as flat sheets or cylinders, into monomolecular protein lattices on artificial surfaces (e. g. silicon wafers, noble metals, plastics) or on Langmuir lipid films and liposomes [4-6]. S-layer proteins from Bacillaceae specifically recognize a distinct type of secondary cell wall polymer (SCWP) as the proper anchoring structure in the rigid cell wall layer [7-10]. The SCWP of *Ly. sphaericus *CCM 2177 consists of disaccharide repeating units that are composed of N-acetyl glucosamine (GlcNAc) and N-acetyl mannosamine (ManNAc). The ManNAc residues carry a pyruvate ketal which endows the polymer chains with a negative net charge [7]. In previous studies, isolated S-layer-specific SCWP was also used as biomimetic linker for the oriented recrystallization of recombinant S-layer fusion proteins incorporating foreign functional sequences, such as antigens, antibodies, ligands, or enzymes on solid supports [5,6].

Due to the intrinsic adjuvant ability of S-layers as well as their capability to display proteins and epitopes on their surface, S-layers are excellent candidates to be used as antigen carriers [11]. Specific immunotherapy (SIT), a method consisting of the consecutive injection of increasing doses of specific allergen to induce specific tolerance in the allergic patient, represent the only causative treatment for Type I allergy [12]. Attempts to improve vaccines for SIT focused on the allergic molecules as well as on adjuvants that support the shift of the Th2-based immune response toward a more balanced phenotype [13]. For this purpose, in recent studies, the gene sequence encoding the major birch pollen allergen Bet v1, was fused with the genes encoding the S-layer protein of *Geobacillus stearothermophilus *ATCC 12980 or *Lysinibacillus sphaericus *CCM 2177 resulting in the recombinant fusion proteins rSbsC/Bet v1 or rSbpA/Bet v1, respectively [14,15]. Both S-layer/allergen fusion proteins contained all relevant Bet v1-specific B and T cell epitopes, but were significantly less efficient to release histamine than rBet v1 [13,16,17]. Further immunological studies showed that both fusion proteins displayed strongly reduced IgE binding capacity than free rBet v1 and promoted the induction of allergen-specific Th0/1 cells and regulatory T cells [13,16,17]. Derived from these results, both recombinant S-layer/allergen fusion proteins can be considered as promising candidates for the development of vaccines for specific immunotherapy of Type I allergies.

In previous studies, gram-negative *E. coli *was used for heterologous expression of rSbsC/Bet v1 or rSbpA/Bet v1, respectively. Moreover, a very material and time consuming purification procedure was performed to remove endotoxin which was associated with the isolated S-layer/allergen fusion proteins [14,15]. In immunological applications, the presence of small amounts of endotoxins, also called lipopolysaccharides (LPS), co-purified with recombinant protein can cause fever, tissue injury, endotoxin shock syndrome or the activation of non-specific immune response in macrophages or B cells [18]. Due to the fact that endotoxin removal techniques belong to the most difficult tasks in downstream processes during protein purification, the establishment of a gram-positive endotoxin-free expression system for S-layer/allergen fusion proteins was required.

The choice of an appropriate host and suitable production conditions is crucial for the downstream processing of a pharmaceutical-grade product [19]. In contrast to the gram-negative *E. coli, B. subtilis *is considered as a GRAS (generally recognized as safe) organism [20]. The reason for the usage of *B. subtilis *as cell factory for the production of proteins with biotechnological application potential, are a) its ability to secrete functional extracellular proteins directly to the culture medium and b) the accumulation of information concerning its transcription and translation mechanisms, genetic manipulation and large scale fermentation which has been acquired during the last years [21]. A further advantage for expression of recombinant S-layer/allergen fusion proteins can be seen in the fact that *B. subtilis *strains carry no S-layer on their cell surface.

In the present study, the strain *B. subtilis *1012, a non-pathogenic organism with naturally high secretory capacity was used as host for expression of the recombinant S-layer/allergen fusion protein rSbpA/Bet v1. *E. coli - B. subtilis *shuttle vectors pHT01, for intracellular expression, and pHT43, carrying the coding region for the signal peptide of the amyQ gene encoding a *Bacillus amyloliquefaciens *α-amylase fused to the Shine-Dalgarno sequence for extracelluar expression, were chosen.

## Results

### Construction of *E. coli-B. subtilis *shuttle vectors pHT01 and pHT43 carrying the chimaeric gene encoding the S-layer/allergen fusion protein rSbpA/Bet v1 as well as transformation of *B. subtilis *1012

The *E. coli-B. subtilis *shuttle vectors pHT01 and pHT43 allow expression of recombinant proteins within the cytoplasm, where pHT43 directs the target protein into the medium. Both vectors are based on the σ^A^-dependent promoter preceding the *groE *operon of *B. subtilis *which has been converted into an IPTG-inducible promoter by addition of the lac operator. For construction of the *B. subtilis *expression vectors, the chimaeric gene *sbpA/bet v1 *was cloned into the cloning sites *Aat*II and *Sma*I of plasmid pHT01 or pHT43, respectively.

**Figure 1:**
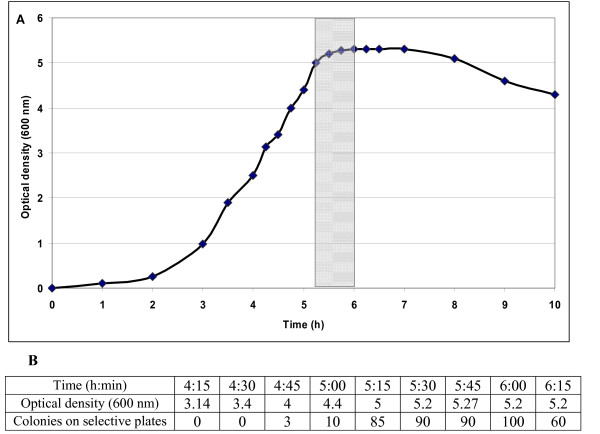
**Estimation of the point in time for development of maximal natural competence of *B. subtilis *1012**. A) Growth curve of *B. subtilis *1012 which was grown under vigorous shaking at 37°C in HS medium at 37°C. After 5 h 15 min at an optical density of 5, the culture reached the early stationary phase. From a cultivation time of 4 h 15 min to 6 h 15 min, aliquots were taken at 15-min time intervals. Transformation of aliquots from each point in time with the reference plasmid pHT43 showed that *B. subtilis *developed natural compentence in the period from 5 h 15 min to 6 h of cultivation (grey column). B) Estimation of transformation competence of *B. subtilis *1012 at different growth phases. After transformation with plasmid pHT43, the number of colonies on selective plates containing chloramphenicol revealed that maximal transformation rates could be obtained by using aliquots taken after 6 h of cultivation at an optical density of 5.2.

For transformation, *B. subtilis* 1012 was grown under vigorous shaking in HS medium at 37°C and the growth curve was recorded (Figure 1A). After 5 h 15 min at an optical density of 5, the culture reached the early stationary phase. From a cultivation time of 4 h 15 min to 6 h 15 min, aliquots were taken at 15-min intervals and stored at -80°C. For estimation of the aliquots yielding high level competent cells, one aliquot from each point in time was checked by transformation with the reference plasmid pHT43. *B. subtilis *1012 developed competence in the period from 5 h 15 min to 6 h of cultivation (Figure 1A, grey column). In Figure 1B, the transformation competence of *B. subtilis *1012 at different growth rates is shown. As estimated by counting of colonies on selective plates containing chloramphenicol, maximal transformation rates could be obtained by using aliquots taken after 6 h of cultivation at an optical density of 5.2 (Figure 1B). For transformation with the plasmids pHT01/*sbpA/bet v1* or pHT43/*sbpA/bet v1*, respectively, *B. subtilis *1012 cryo cultures of maximal competence were used and positive *B. subtilis *transformants were detected on selective LB plates containing chloramphenicol.

### Intracellular and extracellular expression of the S-layer/allergen fusion protein rSbpA/Bet v1 by *B. subtilis *1012 and immunoblot analysis

**Figure 2:**
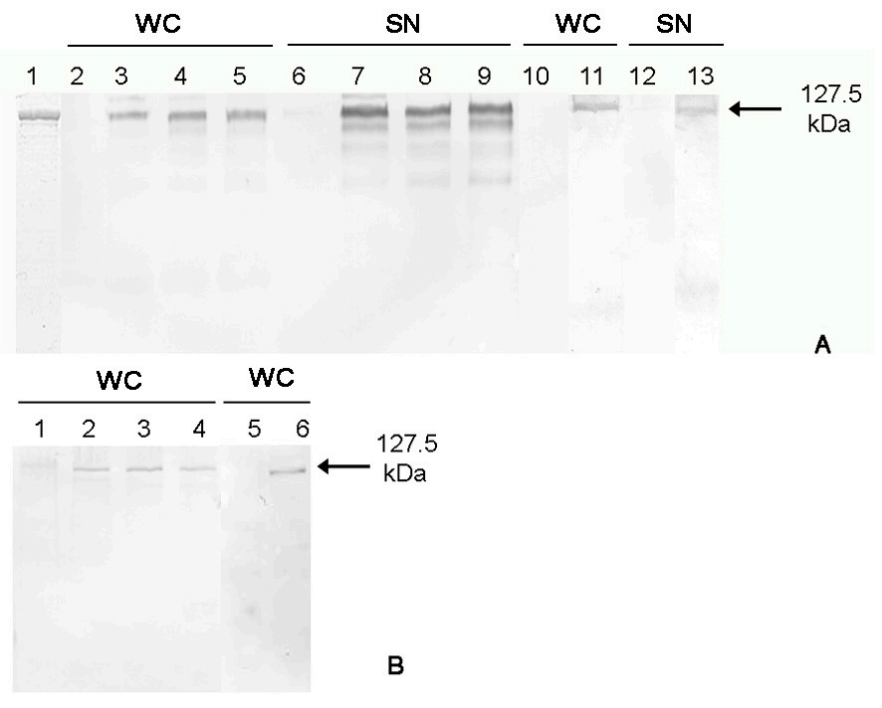
**Intra- and extracelluar expression of the S-layer/allergen fusion protein rSbpA/Bet v1 by *B. subtilis *1012**. (A) Extracellular expression. Immunoblot analysis using the polyclonal rabbit antiserum raised against the S-layer protein SbpA. Purified S-layer fusion protein rSbpA/Bet v1 isolated from *E. coli *BL21Star(DE3) used as positive control (lane 1), SDS-extracted whole cells of *B. subtilis *1012 containing plasmid pHT43/*sbpA/bet v1 *before (lane 2) and 1 h, 2 h and 3 h after induction of fusion protein expression (lane 3, 4 and 5). Supernatant fraction of the *B. subtilis *1012 cell culture before (lane 6) and 1 h, 2 h, 3 h after induction of expression (lane 7, 8 and 9). Immunoblot analysis using the monoclonal antibody BIP1 raised against Bet v1. SDS-extracted whole cells of *B. subtilis *1012 containing plasmid pHT43/*sbpA/bet v1 *before (lane 10) and 2 h after induction of fusion protein expression (lane 11). Supernatant fraction of the *B. subtilis *1012 cell culture before (lane 12) and 2 h after induction of expression (lane 13). WC: whole cells, SN: supernatant (B) Intracellular expression. Immunoblot analysis using the polyclonal rabbit antiserum raised against the S-layer protein SbpA. SDS-extracted whole cells of *B. subtilis *1012 containing plasmid pHT01/*sbpA/bet v1 *before (lane 1) and 1, 2, 3 h after induction of fusion protein expression (lane 2, 3 and 4). Immunoblot analysis using the monoclonal antibody BIP1 raised against Bet v1. SDS-extracted whole cells of *B. subtilis *1012 containing plasmid pHT01/*sbpA/bet v1 *before (lane 5) and 2 h after induction of fusion protein expression (lane 6).

To study the expression as well as the secretion of rSbpA/Bet v1 by *B. subtilis *1012 harbouring plasmid pHT43/*sbpA/bet v1*, samples of SDS-extracted whole cells and culture supernatants were taken before and 1 h, 2 h, and 3 h after induction of expression and examined by SDS-PAGE (Figure 2A). Immunoblot analysis using a polyclonal antiserum raised against the S-layer protein SbpA confirmed the occurrence of the SbpA portion in the fusion protein in the cell pellet (Figure 2A, lane, 3-5) as well as in the supernatant (Figure 2A, lanes 7-9) by a protein band migrating at 127.5 kDa. As shown in Figure 2A, lanes 11 and 13, immunoblots performed with the monoclonal antibody BIP1 raised against Bet v1 detected the Bet v1 portion in the protein band with the size of 127.5 kDa on the SDS gel. Recombinant rSbpA/Bet v1 derived from expression in *E. coli *produced in a former study [15] and subjected to SDS-PAGE as a positive control showed a protein band of 127.5 kDa (Figure 2A, lane 1). In samples taken before induction of expression, no protein band could be detected by using anti SbpA-antiserum (Figure 2A, lane 2 and 6) or BIP1 antibody (Figure 2A, lanes 10 and 12). The intensity of the detected protein bands revealed that, after induction of expression in *B. subtilis *1012 carrying pHT43/*sbpA/bet v1*, the main part of the fusion protein was secreted into the culture medium. A faint protein band migrating at 127.5 kDa could be detected in cell pellets of *B. subtilis *1012 (Figure 2A, lanes 3-5 and lane 11). Electron microscopical investigation of thin sections of *B. subtilis *1012 cells harvested 3 h after induction of *rsbpA/bet v1 *expression showed no S-layer self-assembly products in the cell cytoplasm (data not shown).

Investigation of intracellular expression of rSbpA/Bet v1 by *B. subtilis *1012 carrying pHT01/*sbpA/bet v1* done by immunoblotting of SDS-extracted cell pellets with anti-SbpA antiserum (Figure 2B, lanes 2-4) or BIP1 (Figure 2B, lane 6) showed only a faint protein band of 127.5 kDa. Extension of expression time until over night did not result in higher yield of rSbpA/Bet v1 (data not shown). No S-layer fusion protein could be found before induction of expression (Figure 2B, lanes 1 and 5) or in the culture supernatant (data not shown). By comparing the growth curves of induced and non-induced *B. subtilis *cells carrying pHT43/*sbpA/bet v1 *and pHT01/*sbpA/bet v1*, respectively, it could be demonstrated that expression of rSbpA/Bet v1 did not affect growth of *B. subtilis *(data not shown).

### TEM analysis of *B. subtilis *1012 cells after expression and secretion of the S-layer/allergen fusion protein rSbpA/Bet v1

**Figure 3:**
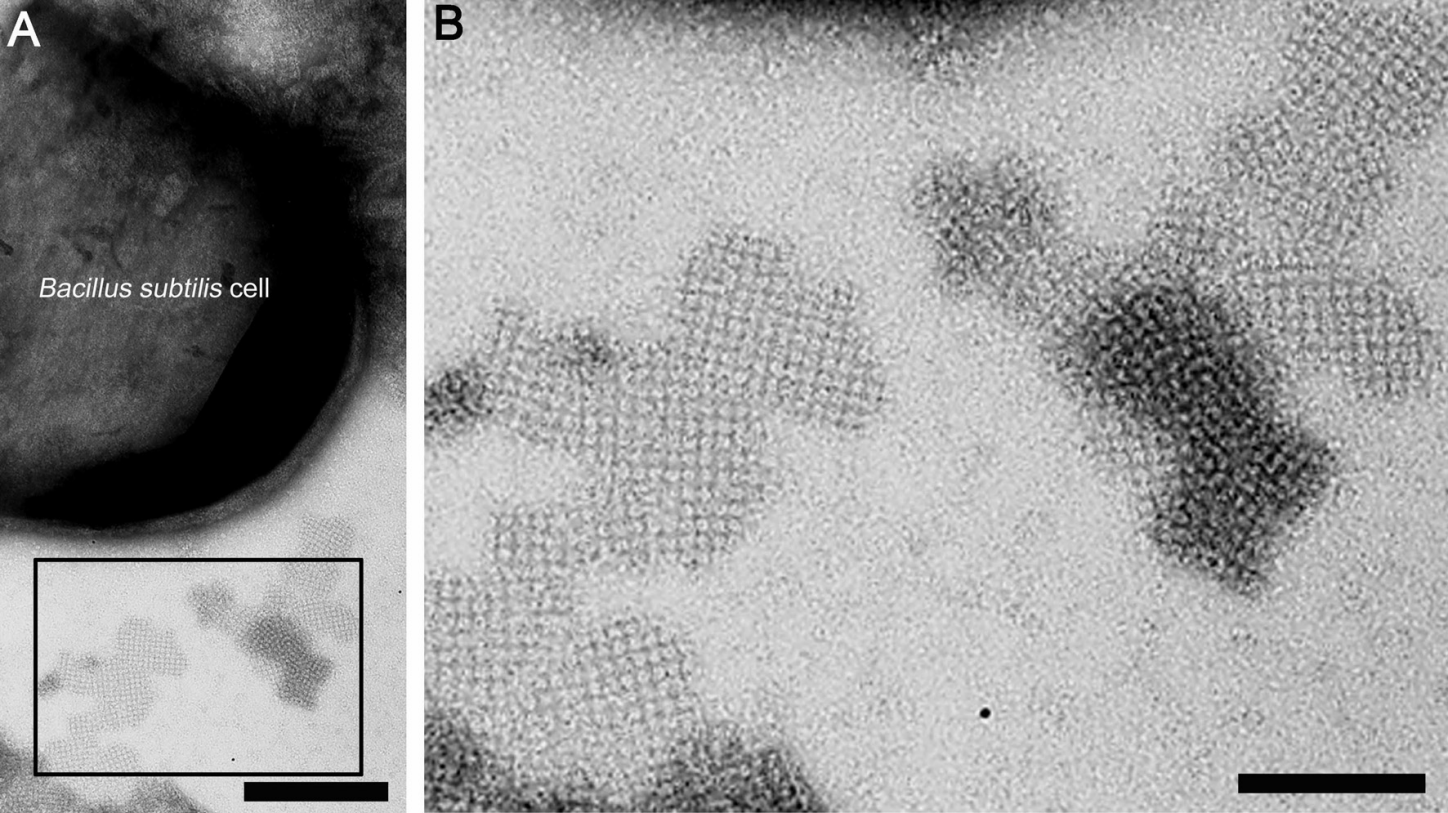
**Electron micrograph of a negatively stained preparation of *B. subtilis *1012 cells carrying plasmid pHT43/*sbpA/bet v1, *3 h after induction of *rsbpA/bet v1 *expression**. (A) After secretion into the culture medium, the heterologously produced S-layer/allergen fusion protein did not recrystallize on the cell surface of *B. subtilis *1012 but was able to form self-assembly products with a maximum size of 1 μm which clearly exhibited the square S-layer lattice symmetry. Bar = 100 nm (B) Detailed view on the ultrastructure of the rSbpA/Bet v1 self-assembly products which show a center-to-center spacing of the morphological unit of 13.1 nm. Bar = 50 nm.

Three hours after induction of expression, an aliquot of the *B. subtilis *cell culture carrying plasmid pHT43/*sbpA/bet v1 *was harvested and subjected to negative staining. Electron microscopical investigation showed that the secreted S-layer/allergen fusion protein did not recrystallize on the cell surface of *B. subtilis *1012, however, in the culture medium, mono- and double layered rSbpA/Bet v1 self-assembly products could be identified (Figure 3A). Ultrastructural investigation of the self-assembly products revealed a size of up to 1 μm and a square S-layer lattice symmetry with a center-to-center spacing of the subunits of 13.1 nm (Figure 3B). These results indicated that the amount of fusion protein which is visible in the sedimented cell fraction on the SDS-gel (Figure 2A, lanes 3-5) is not located in the cell cytoplasm of the host cell. As derived from TEM analysis, the protein band can be attributed to secreted and assembled rSbpA/Bet v1 which was co-sedimented with *B. subtilis *cells by centrifugation.

### Recrystallization of the S-layer/allergen fusion protein on solid supports, immunogold-labelling and electron microscopical investigation

**Figure 4:**
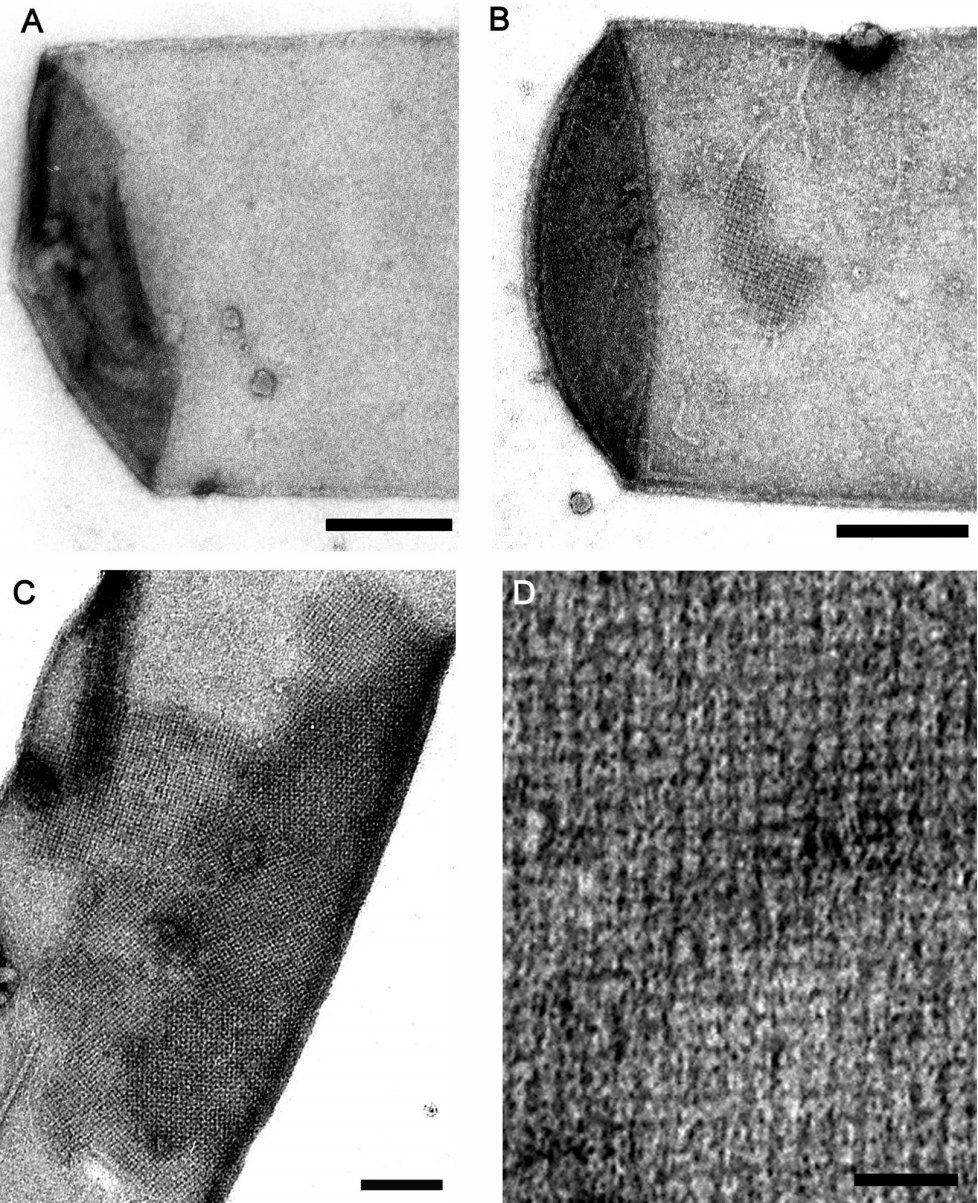
**Electron micrograph of negatively stained preparations describing concentration and purification of the S-layer/allergen fusion protein rSbpA/Bet v1 from *B. subtilis *culture medium by recrystallization on solid supports**. (A) Peptidoglycan-containing sacculus of *Ly. sphaericus *CCM 2177 without S-layer lattice. Bar = 100 nm (B) Peptidoglycan-containing sacculus of *Ly. sphaericus *CCM 2177 after first incubation with *B. subtilis *1012 culture medium containing secreted rSbpA/Bet v1 monomers. A patch of recrystallized rSbpA/Bet v1 on the surface exhibiting the square lattice structure is clearly visible. Bar = 100 nm (C) Peptidoglycan-containing sacculus of *Ly. sphaericus *CCM 2177 after repeated incubation with *B. subtilis *1012 culture medium containing secreted rSbpA/Bet v1 monomers. Bar = 100 nm (D) The ultrastructure of the closed rSbpA/Bet v1 monolayer on the solid support in detail. Bar = 50 nm.

To isolate and concentrate the amount of secreted soluble rSbpA/Bet v1 fusion protein from the *B. subtilis *culture supernatant, the high specific binding mechanism between the conserved S-layer homology (SLH) domain at the N-terminus of the S-layer protein and the negatively charged secondary cell wall polymer in the rigid cell wall layer of *Ly. sphaericus *CCM 2177 was exploited. For that purpose, the wild-type S-layer protein SbpA was completely extracted from the surface of cell wall fragments of *Ly. sphaericus *CCM 2177 by incubation with a chaotropic agent. Three hours after induction of *rsbpA/bet v1 *expression, the obtained plain peptidoglycan-containing sacculi of *Ly. sphaericus *CCM 2177 (Figure 4A) were added to an aliquot of the *B. subtilis *1012/pHT43/*sbpA/bet v1 *culture supernatant containing secreted rSbpA/Bet v1 monomers. The mixture was adjusted to 10 mM CaCl_2 _because previous studies revealed that the recrystallization process of the S-layer protein SbpA is dependent on the presence of calcium ions allowing control over lattice formation. Negative staining and electron microscopical investigation revealed small patches of recrystallized fusion protein on the surface of the solid support (Figure 4B). To accumulate the S-layer fusion protein on the sacculi, the incubation procedure was repeated several times (Figure 4C). Finally, peptidoglycan-containing sacculi completely covered with recrystallized S-layer/allergen fusion protein clearly exhibiting the square lattice symmetry could be obtained (Figure 4D).

**Figure 5:**
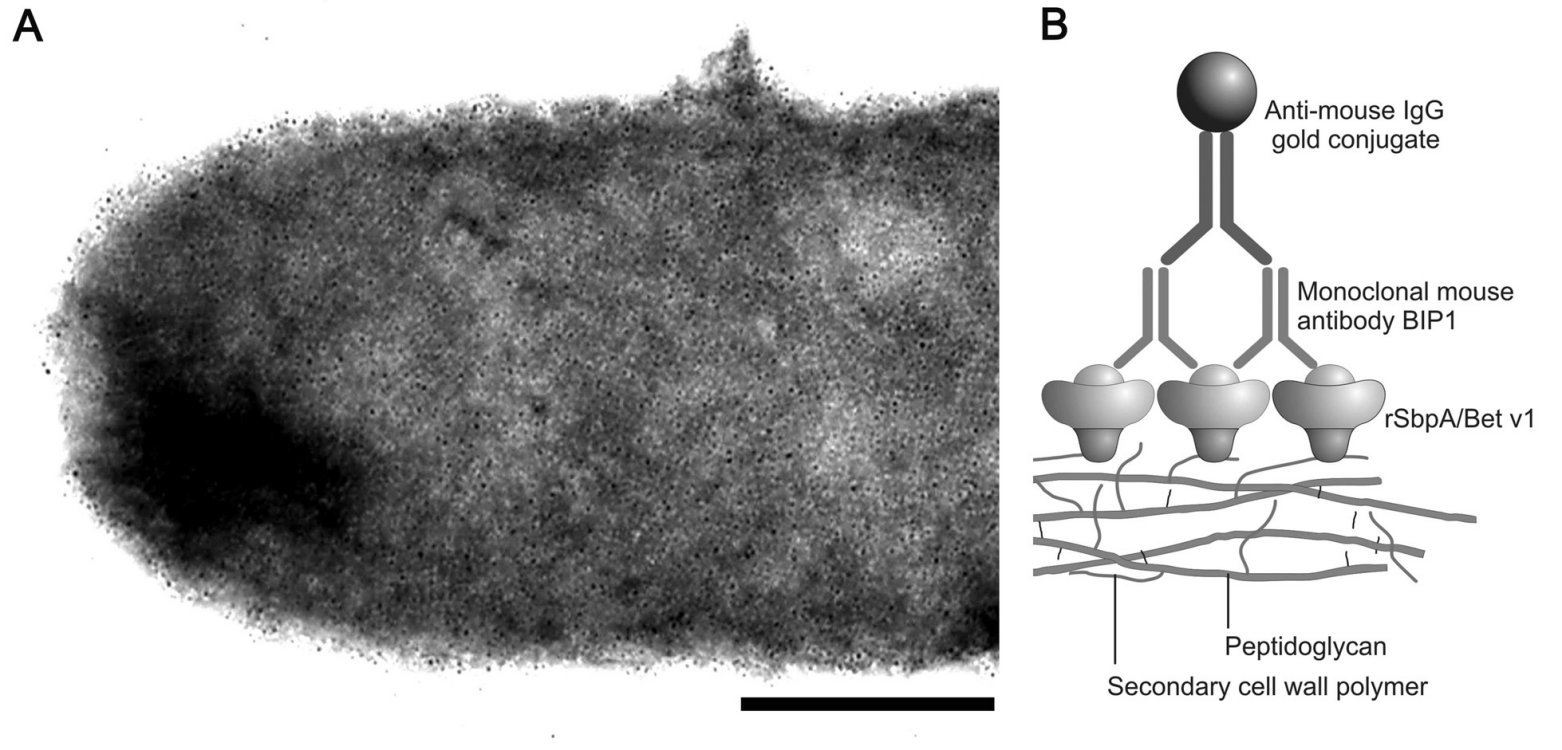
**Immunogold labelling of rSbpA/Bet v1 recrystallized on a solid support**. After production in *B. subtilis *1012, the S-layer/allergen fusion protein rSbpA/Bet v1 recrystallized on peptidoglycan-containing sacculi of *Ly. sphaericus *CCM 2177 was incubated with the monoclonal mouse antibody BIP1 raised against Bet v1. Bound BIP1 was detected by using colloidal gold-labelled anti-mouse IgG. Bar = 100 nm.

For immunogold-labelling of the crystalline monolayer formed by oriented recrystallization of rSbpA/Bet v1 on peptidoglycan-containing sacculi of *Ly. sphaericus *CCM 2177, the recrystallization products were incubated with monoclonal mouse antibody BIP1 which was visualized by an anti-mouse IgG colloidal gold conjugate. As shown in Figure 5, TEM analysis revealed that the surface of the recrystallization products was completely covered with gold particles (5 nm) except small areas where no fusion protein could be found. These results indicated that the Bet v1 portion was located on the outer surface of the S-layer lattice. If samples of rSbpA which were taken as negative control were recrystallized on sacculi of *Ly. sphaericus *CCM 2177, no gold labelling could be observed in negatively stained preparations (data not shown).

### Quantification of the amount of purified rSbpA/Bet v1 after extracelluar expression by *B. subtilis *1012

**Figure 6:**
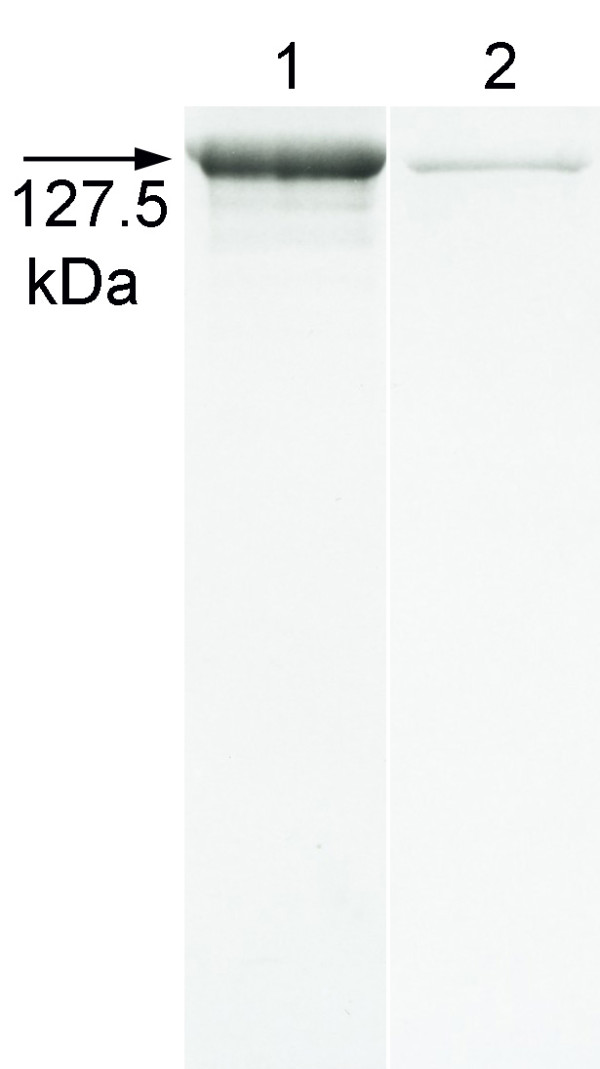
**Quantification of extracelluar expression of rSbpA/Bet v1 by *B. subtilis *1012**. SDS-PAGE analysis of rSbpA/Bet v1 (10 μg) purified from *E. coli *(lane 1) and of rSbpA/Bet v1 isolated from 1 mg peptidoglycan-containing sacculi of *Ly. sphaericus *CCM 2177 after incubation with 20 ml culture supernatant of a *B. subtilis *1012 culture harvested 3 h after induction of expression (lane 2).

For quantification of expression, recrystallization products consisting of rSbpA/Bet v1 (derived from 20 ml culture supernatant) recrystallized on 1 mg peptidoglycan-containing sacculi of *Ly. sphaericus *CCM 2177 were incubated with 2 ml of a chaotrophic agent. After separation of the fusion protein rSbpA/Bet v1 from the peptidoglycan-containing sacculi by centrifugation, the supernatant was dialyzed against distilled water and 10 μl were subjected to SDS-PAGE analysis (Figure 6, lane 2). Furthermore, 10 μl of a 1 mg/ml stock solution (10 μg) of rSbpA/Bet v1 purified from *E. coli *was also subjected to the SDS-PAGE and used as a reference (Figure 6, lane 1). Derived from these data, quantification of the protein concentration of purified rSbpA/Bet v1 after extracelluar expression by *B. subtilis *1012 revealed a protein amount of ~ 50 mg per liter expression medium.

### Reaction of rSbpA/Bet v1 with Bet v1 specific IgE on immunoblots

**Figure 7:**
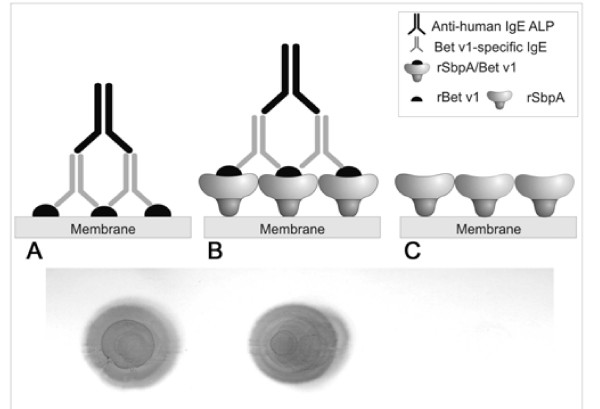
**Dot blot assay indicating the IgE reactivity of rSbpA/Bet v1 fusion protein**. Results indicated that rSbpA/Bet v1 (B) and rBet v1 (A) which was used as a positive control showed strong IgE reactivity when incubated with Bet v1-specific serum samples of birch pollen allergic patients. Recombinant SbpA (C) used as a blank did not show any reactivity.

The functionality of the Bet v1 domain in the fusion protein was demonstrated by binding of IgE to rSbpA/Bet v1 monomers spotted on a nitrocellulose membrane. As shown in a dot blot assay, IgE from a serum sample of patients suffering atopic allergy caused by birch pollen recognized rSbpA/Bet v1 (Figure 7B) and showed a comparable reaction to the positive control, for which rBet v1 was taken (Figure 7A). Recombinant SbpA used as negative control did not show any IgE binding capacity (Figure 7C).

### Biocompatibility tests for investigation of Limulus amebocyte lyste (LAL) reactivity of rSbpA/Bet v1 expressed in *B. subtilis *1012 compared to the S-layer/allergen fusion protein expressed in *E. coli*

LAL reactivity of rSbpA/Bet v1 expressed in gram-positive *B. subtilis *1012 was tested and compared to the endotoxin contamination of the recombinant S-layer/allergen fusion protein isolated from gram-negative *E. coli*. LAL assays revealed a LPS value of 20 EU/ml for rSbpA/Bet v1 after intracellular expression in *E. coli*, isolation from the host cell and purification with gel permeation chromatography (GPC) as well as an endotoxin value of 1-2 EU/ml after a second GPC purification step. In contrast to these results, no endotoxin could be detected in rSbpA/Bet v1 expressed by *B. subtilis *1012 and secreted into the culture medium.

## Discussion

Because of their temperature and pH stability, the removal of bacterial endotoxins becomes more difficult when associated with labile biomolecules, such as proteins [22]. Besides commonly used techniques like for example ultrafiltration [23] and ion exchange chromatography [24], affinity chromatography is reported as an effective method to reduce endotoxin in solutions [25]. Ultrafiltration can be useful to remove endotoxin aggregates [22] but with large proteins like rSbpA/Bet v1 with a molecular weight of 127.5 kDa, used in this study, this method is not effective. Due to the fact, that S-layers are insoluble in buffers usually taken for affinity chromatography, this technique turned out to be unsuitable for endotoxin removal from S-layer preparations. To achieve complete solubilization of S-layer proteins into their constituent subunits, the addition of high concentrated hydrogen-bond-breaking agents (e. g. urea, guanidinium hydrochloride) is required [5,6,26].

In recent years, various studies revealed that non-pathogenic gram-positive *B. subtilis *is an attractive host organism for the expression and secretion of heterologous proteins [19,21,27]. The advantage of secretion of the target protein can be seen in a natural separation of the product from cell components simplifying downstream processing as well as in the provision of better refolding conditions compared to the reducing conditions in the cell cytoplasm [19].

In the present study, with the aim to produce an endotoxin-free S-layer/allergen fusion protein, a gram-positive expression system was developed based on the expression host *B. subtilis *1012 as well as the *E. coli-B. subtilis *shuttle vectors pHT01 or pHT43 carrying the amyQ signal sequence. To estimate the point in time for development of maximal natural competence, the growth curve of *B. subtilis *1012 was recorded and culture aliquots of estimated maximal competence were used for transformation with the plasmids carrying a chimaeric gene encoding rSbpA/Bet v1. In the case of *B. subtilis *1012 cells carrying pHT43/*sbpA/bet v1*, comparison of the amount of rSbpA/Bet v1 in whole cells and in the supernatant by SDS-PAGE revealed that the main part of the produced fusion protein was secreted into the culture medium from the beginning to the end of the stationary growth phase. From these results, it can be concluded that the signal peptide of the *B. subtilis *α-amylase works well for S-layer fusion proteins and the high molecular weight of the target protein did not interfere with the secretion process. Additionally, it could be demonstrated that the growth rate of *B. subtilis *was not affected by the induction of *rsbpA/bet v1 *expression.

In [28], Howorka and co-workers reported the self-assembly formation of SbsA, the S-layer protein of *G. stearothermophilus *PV72/p6 in the course of autolysis after expression in *B. subtilis*. This expression system based on the xylose-inducible multicopy plasmid pSTR5 carrying the *sbsA *gene as well as the signal peptide of the S-layer protein SbsA showed high level intracellular expression but rSbsA was retained in the *B. subtilis *peptidoglycan layer and therefore was poorly secreted into the ambient cellular environment. In former studies concerning the heterologous expression in *E. coli*, it could be demonstrated that the recombinant S-layer proteins SbsA of *G. stearothermophilus *PV72/p6, SbsB of *G. stearothermophilus *PV72/p2 or SbsC from *G. stearothermophilus *ATCC 12980, respectively, formed self-assembly products in the cell cytoplasm directly after expression [29-31]. In contrast to these results, recombinant SbpA as well as the rSbpA/Bet v1 fusion protein did not recrystallize in the *E. coli *cell cytoplasm [15]. In view of the desired secretion of the SbpA/allergen fusion protein after expression in *B. subtilis*, in the present study, this fact turned out to be an advantage. As shown by electron microscopical investigation of thin sections of *B. subtilis *1012 cells harvested 3h after induction of expression, no rSbpA/Bet v1 self-assembly products were visible in the cell cytoplasm of the host cells. Nearly 80% of rSbpA/Bet v1 secreted into the culture medium were soluble but electron microscopical investigation of *B. subtilis *culture samples taken 3 h after induction revealed that up to 20% of secreted fusion protein formed self-assembly products. According to results obtained by [28], no rSbpA/Bet v1 S-layer lattice could be observed on the cell surface of *B. subtilis*. However, for *B. subtilis *cells containing plasmid pHT01/*sbpA/bet v1*, just a weak intracellular expression of fusion protein could be observed. In order to increase the yield of expression, it could be conducive to replace the *B. subtilis *P_grac _promoter by one of the native *sbpA *promoters identified in the upstream region of the *sbpA *gene of *Ly. sphaericus *CCM 2177 in future studies[15].

Secreted rSbpA/Bet v1 could be concentrated by exploiting the specific interaction between the conserved N-terminus of SbpA in the fusion protein and a negatively charged pyruvylated SCWP which is located in the peptidoglycan-containing layer of *Ly. sphaericus *CCM 2177. In accordance to former studies performed with the S-layer/allergen fusion protein isolated from *E. coli *[14,15], rSbpA/Bet v1 expressed and secreted by *B. subtilis *1012 showed excellent recrystallization properties in suspension and on solid supports. By immunoblotting, the binding capacity of purified rSbpA/Bet v1 for IgE from serum samples of birch pollen allergic patients could be demonstrated.

## Conclusions

In the present study for the first time, a pyrogen-free recombinant S-layer/allergen fusion protein required for vaccine development was produced using a gram-positive expression system based on *B. subtilis *1012. The usage of an *E. coli-B. subtilis *shuttle vector carrying the sequence encoding the signal peptide of *B. subtilis *α-amylase confers on the gram-positive organism the ability to secrete the expressed S-layer/allergen fusion protein rSbpA/Bet v1 directly into the culture medium. Structural and immunological investigations of the obtained fusion protein revealed that rSbpA/Bet v1 was endotoxin-free, showed excellent recrystallization properties and immune reactivity.

## Methods

### Bacterial strains, plasmids and oligonucleotide primers

Bacterial strains, plasmids and oligonucleotides used in this study were listed in Table 1.

**Table 1:** Bacterial strains, plasmids and oligonucleodide primers used in this study.

Description	Contents	Reference/source
Strains		
*E. coli *TG1	supE thi-1 Δ(lac-proAB) Δ(mcrB-hsdSM)5(rK- mK-) [F' traD36 proAB lacIqZΔM15	Stratagene
*B. subtilis *1012	*leuA*8, *metB*5, *hsrM*1, non*A*	CatchMaps BV, MoBiTec
Plasmids		
pET28a/*sbpA/bet v1*	Cloning of *rsbpA/bet v1*	[15]
pHT01	Amp^r^, Cm^r^, P*grac *promoter (consisting of groEpromotor, the lacO operator and the gsiBSD sequence, ColE1 ori,, lacI repressor, *E. coli*/*B. subtilis *shuttle vector	MoBiTec
pHT43	see pHT01 + amyQ signal sequence	MoBiTec
pHT01/*sbpA/bet v*1	Expression of rSbpA/Bet v1 in cytoplasm of *B. subtilis *1012	This study
pHT43/*sbpA/bet v1*	Expression of rSbpA/Bet v1 in cytoplasm of *B. subtilis *1012 followed by subsequent secretion into the culture medium	This study
Primers*		
SbpA/AatII/forward	5'-cgc**gacgtc**gcgcaagtaaacgactataacaaatc-3'	This study
Bet v1/SmaI/reverse	5'-tcc**cccggg**ttagttgtaggcatcggagtgtg-3'	This study

*Bold nucleotide sequences indicate restriction endonuclease sites.

### Cloning of the chimaeric gene encoding rSbpA/Bet v1 in pHT01 and in pHT43

For amplification of the gene encoding the S-layer/allergen fusion protein rSbpA/Bet v1 (devoid of the sequence encoding the SbpA signal peptide), the plasmid pET28a (Invitrogen) carrying the chimaeric gene *sbpA/bet v1 *derived from a previous study [15] was isolated from *E. coli *TG1 and used as template. PCR amplifications were performed as described in [32]. The oligonucleotide primer SbpA/AatII/forward as well as the primer Bet v1/SmaI/reverse (all listed in Table 1) introduced the cloning sites *Aat*II and *Sma*I at the 5' and 3'-end, respectively. General procedures for DNA manipulations were carried out as reported by [33]. Cloning of pHT01 (MoBiTec) and pHT43 (MoBiTec) carrying the gene encoding rSbpA/Bet v1 in *E. coli *TG1 (Stratagene) was done as described in [32]. For selection of positive transformants, the constructs were grown in 3 ml Luria-Broth Base medium (Invitrogen) at 37°C and carbenicillin (Fluka) was added to a final concentration of 50 μg/ml. Finally, plasmids pHT01/*sbpA/bet v1 *and pHT43/*sbpA/bet v1 *were re-isolated from *E. coli *TG1 by using QIAprep Spin Miniprep Kit (Qiagen) and stored at -20°C. The correct construction of the vectors was confirmed by sequencing.

### Preparation of competent *B. subtilis *1012 cells

*B. subtilis *1012 (CatchMaps BV) was grown overnight at 37°C in a test tube on 5 ml HS medium (66.5 ml A. dest., 10 ml 10 × S-base (Spizizen's salt), 2.5 ml 20% (w/v) glucose, 5 ml 0.1% (w/v) L-tryptophan, 1 ml 2% (w/v) casein, 5 ml 10% (w/v) yeast extract (Difco), 10 ml 8% (w/v) arginine, 0.4% histidine) under shaking at 200 rpm. Subsequently, 0.5 ml of the overnight culture were diluted in 50 ml HS medium in a 500-ml flask, incubated under shaking at 200 rpm at 37°C and the growth curve was recorded. When cells reached the stationary growth phase, samples of 10 ml were taken at 15-min time intervals. Each aliquot was mixed with 1 ml 87% glycerol and incubated for 15 min on ice. After fractionating into 1-ml aliquots, all samples were frozen in liquid nitrogen and stored at -80°C. For estimation of the aliquots yielding high level competent cells, one aliquot from each point in time was checked by transformation with a reference plasmid pHT43 (MoBiTec) without insert.

### Transformation of *B. subtilis *1012

For transformation of *B. subtilis *1012, the slightly modified protocol of [34] was used. Cryo cultures of competent *B. subtilis *1012 cells were thawed at 37°C, one aliquot (1 ml) was used to inoculate 20 ml HS medium and the culture was shaken at 200 rpm in a 30°C-water bath for 2 h. Aliquots of 1 ml were put into a glass tube, 10 μl of 0.1 M EGTA, pH 7.2 were added and the mixture was incubated for 5 min at room temperature. Plasmid DNA (3 μg) pHT01/*sbpA/bet v*1 or pHT43/*sbpA/bet v1*, respectively, was added and the sample was incubated for 2 h at 37°C while shaking at 200 rpm. For detection of positive *B. subtilis *transformants, cells were plated on selective LB plates containing chloramphenicol at a final concentration of 10 μg/ml.

### Expression of the S-layer/allergen fusion protein in *B. subtilis *1012, preparation of cellular and supernatant fractions and immunoblot analysis

*B. subtilis *1012 cells carrying the plasmids pHT01/*sbpA/bet v*1 or pHT43/*sbpA/bet v1*, respectively, were grown in 3 ml LB medium containing chloramphenicol at a final concentration of 10 μg/ml in a test tube under shaking at 200 rpm at 37°C overnight. Subsequently, 25 ml LB medium were inoculated with 250 μl of the culture (dilution 1:100) containing chloramphenicol at a final concentration of 10 μg/ml and incubated under shaking at 200 rpm at 37°C. At an optical density of 2 at 600 nm, expression of rSbpA/Bet v1 was induced by addition of IPTG at a final concentration of 1 mM. Samples (1.5 ml) of each *B. subtilis *1012 culture were taken before as well as 1 h, 2 h, 3 h and 18 h after induction of expression of rSbpA/Bet v1. The supernatant and cellular fractions were separated by centrifugation at 20,000 × g for 30 min at 4°C. Cell pellets were incubated with lysozyme (final concentration 1 mg/ml) for 5 min at 37°C before addition of Laemmli buffer [35]. Preparation of samples for SDS-PAGE analysis was carried out as described by [35].

Immunoblotting with a polyclonal rabbit antiserum raised against the S-layer protein SbpA of *Ly. sphaericus *CCM 2177 was performed as described previously [15]. The monoclonal mouse anti-Bet v1 antibody BIP1 was used to check to presence of Bet v1 epitopes in the fusion protein rSbpA/Bet v1 expressed in *B. subtilis *1012 as well as in the fusion protein rSbpA/Bet v1 expressed in *E. coli *(derived from a former study [15]), which was used as a positive control. For that purpose, after separation of the proteins on SDS gels and blotting to a nitrocellulose membrane (Potran; Schleicher and Schüll), blocking with 0.2% (Blocking Reagent CA AppliChem, Germany) in Tris-buffered saline solution (0.1 M TBS, pH 7.2) was applied for 18 h at 4°C. The membrane was then incubated with BIP1 (diluted 1:100 in blocking solution) for 1.5 h at room temperature and washed three times with wash solution (0.1 M TBS containing 0.5% Tween20) and once with 0.1 M TBS. Subsequently, the membrane was incubated with anti-mouse IgG alkaline phosphatase conjugate (Sigma; diluted 1:5,000 in blocking solution) for 1 h at room temperature. After washing steps as described above, bound antibody was detected by incubation with 5-bromo-4-chloro-3-indolyl phosphate and nitro blue tetrazolium chloride (BCIP/NBT; Roche) as precipitating chromogenic substrate.

### Electron microscopical investigation of *B. subtilis *1012 cells after expression and secretion of the S-layer/allergen fusion protein

For electron microscopical examination of *B. subtilis *1012 cells containing pHT43/*sbpA/bet v1 *harvested 3 h after induction of expression, 30 μl of cell culture were transferred onto a 300-mesh Formvar-coated copper grid which was stabilized by vacuum deposition of carbon and rendered hydrophilic by glow discharge [36]. For chemical fixation of the sample, the grid was floated on a drop of 2.5% glutaraldehyde in 0.1 M sodium cacodylate buffer, pH 7.0 for 15 min at room temperature. After 3 washing steps with distilled water, negative staining was done by incubation of the grid on a drop of 1% uranyl acetate for 5 min at room temperature [36]. Fixation, embedding as well as ultrathin-sectioning of *B. subtilis *1012 cells harvested 3 h after induction of *rsbpA/bet v1 *expression was performed as described in a previous study. Electron micrographs were taken with a Philips CM12 transmission electron microscope (Philips Eindhoven, The Netherlands) operated at 80 kV in low-dose mode [15].

### Peparation of peptidoglycan-containing sacculi of *Ly. sphaericus *CCM 2177

*Ly. sphaericus *CCM 2177 (Czech Collection of Microorganism, Brno, Czech Republic) was grown in continuous culture in nutrient broth medium at 30°C at a dilution rate of 0.16 h^-1 ^as described in a previous study [7]. Preparation of cell wall fragments of *Ly. sphaericus *CCM 2177 was done according to [37]. For preparation of peptidoglycan-containing sacculi, cell wall fragments of *Ly. sphaericus *CCM 2177 were incubated twice with 5 M guanidine hydrochloride (GHCl) in 50 mM Tris/HCl, pH 7.2 for 20 min at 4°C for complete removal of the wild-type S-layer protein SbpA. Subsequently, the remaining peptidoglycan pellet was washed four times with 50 mM Tris/HCl, pH 7.2, suspended in 15 ml of distilled water, frozen at -20°C, and lyophilized.

### Purification and concentration of the fusion protein rSbpA/Bet v1 by recrystallization on solid supports, immunogold-labelling and investigation by electron microscopy

For isolation and concentration of the secreted fusion protein rSbpA/Bet v1, 3 h after induction of expression, 1 mg lyophilized peptidoglycan-containing sacculi of *Ly. sphaericus *CCM 2177 were added to a 5-ml aliquot of the *B. subtilis *1012/pHT43/*sbpA/bet v1 *culture supernatant. To induce the recrystallization process of the fusion protein on the surface, the mixture was adjusted to 10 mM CaCl_2 _and incubated by shaking at 4°C overnight. Subsequently, the suspension was centrifuged at 20,000 × g at 4°C for 10 min and the pellet was incubated again with a new 5-ml aliquot of supernatant of the *B. subtilis *1012/pHT43/*sbpA/bet v1 *culture. The procedure was repeated 2 times and the pellet containing recrystallization products was subsequently applied for negative staining and electron microscopical investigation performed as described above.

Immunogold-labelling was done by incubation of the samples with monoclonal mouse antibody BIP1 raised against Bet v1 for 2 h at room temperature. After centrifugation of the suspension at 16,000 × g for 10 min at 4°C and 3 washing steps with 0.15 M TBS buffer, the pellet was suspended in 30 μl anti-mouse IgG-colloidal gold (Sigma) and incubated for 60 min at room temperature. Unbound gold-labelled antibody was removed by centrifugation under conditions described above and three washing steps with 0.15 M TBS buffer. The pellet was resuspended in 20 μl distilled water and subjected to negative staining. As a control, the whole procedure was also performed by using recrystallization products consisting of rSbpA (recombinantly produced in *E. coli *in a previous study [15] which was recrystallized on peptidoglycan-containing sacculi of *Ly. sphaericus *CCM 2177.

### Quantification of the amount of purified rSbpA/Bet v1 after extracelluar expression by *B. subtilis *1012

For quantification of expression, recrystallization products (produced as described above) consisting of rSbpA/Bet v1 (derived from 20 ml culture supernatant) recrystallized on 1 mg peptidoglycan-containing sacculi of *Ly. sphaericus *CCM 2177 were incubated with 2 ml of 5 M GHCl in 50 mM Tris/HCl, pH 7.2 for 10 min at 4°C. The fusion protein rSbpA/Bet v1 was separated from the peptidoglycan-containing sacculi of *Ly. sphaericus *CCM 2177 by centrifugation of the samples at 14,000 rpm for 15 min at 4°C. The supernatant was dialyzed against distilled water for 16 h at 4°C and 10 μl were subjected to SDS-PAGE analysis. To estimate the concentration of the fusion protein in the dialyzed supernatant, 10 μl of a 1 mg/ml stock solution (10 μg) of rSbpA/Bet v1 purified from *E. coli *was also subjected to the SDS-PAGE and used as a reference.

### Immuno dot blot for detection of IgE reactivity of rSbpA/Bet v1 by with serum samples from birch pollen allergic patients

For investigation of IgE reactivity, rSbpA/Bet v1 fusion protein as well as rSbpA acting as blank and rBet v1 used as positive control (both proteins derived from [15]) were spotted onto a nitrocellulose membrane which was subsequently incubated with sera from patients suffering from birch pollen allergy at 4°C for 16 h. The procedure was followed by incubation with monoclonal anti-human IgE alkaline phosphatase conjugate (Sigma; diluted 1:6,000) and detection with BCIP/NBT was done as described above.

### Biocompatibility test for investigation of LAL reactivity of rSbpA/Bet v1 expressed in *B. subtilis *1012

Detection and quantification of gram-negative bacterial endotoxins of 100 μl culture medium containing rSbpA/Bet v1 secreted by *B. subtilis *1012 during 3 h of expression was performed following the manufacturer's instructions (Limulus Amebocyte Lysate, PYROCHROME^®^, U.S.). Results were compared with those obtained from LAL-tests carried out with rSbpA/Bet v1 which was expressed in *E. coli *BL21Star(DE3) (Invitrogen) and purified as described in a former study [15].

## Competing interests

The authors declare that they have no competing interests.

## Authors' contributions

NI conceived and supervised the whole study, carried out the molecular genetic work, established protocols for transformation, protein purification and recrystallization experiments, performed TEM analysis as well as immunoblotting and drafted the manuscript. CTS performed DNA cloning, transformation and expression in *B. subtilis *1012. BB provided expertise in immunological studies. UBS and EME contributed ideas for performing the experiments, professional support, and helpful suggestions for improving the manuscript.

All authors read and approved the final manuscript.
